# New Biobased Plasticizers for PVC Derived from Saturated Dimerized Fatty Acids

**DOI:** 10.3390/ma18092155

**Published:** 2025-05-07

**Authors:** Patryk Dziendzioł, Sylwia Waśkiewicz, Katarzyna Jaszcz

**Affiliations:** 1 Department of Physical Chemistry and Technology of Polymers, Faculty of Chemistry, Silesian University of Technology, M. Strzody 9, 44-100 Gliwice, Poland; patryk.dziendziol@polsl.pl; 2 Department of Development, R&D of Grupa Azoty Zakłady Azotowe Kędzierzyn S.A., Mostowa 30A Street, 47-220 Kędzierzyn-Koźle, Poland; 3 Joint Doctoral School, Silesian University of Technology, Akademicka 2A, 44-100 Gliwice, Poland

**Keywords:** polymeric plasticizer, biobased, dimerized fatty acid, dry blend, compatibility

## Abstract

Phthalates are compounds widely used as very effective plasticizers of PVC. Unfortunately, they are also widely known to be endocrine disruptors and are detrimental to human health and the environment. For this reason, environmentally friendly plasticizers are being intensively sought after in response to the market needs in the context of sustainable development and legislative changes regarding the use of phthalates. Our research presents an innovative approach to addressing this problem. In this paper, we propose new biobased oligoesters as non-toxic and harmless plasticizers of poly(vinyl chloride). New plasticizers were obtained by polyesterification of saturated dimerized fatty acid (DFA), adipic acid (ADA), triethylene glycol (TEG), and 2-ethylhexanol (2-EH), and were characterized by nuclear magnetic resonance, size exclusion chromatography, and viscosity analyses. The compatibility of the obtained oligoesters with PVC was determined using the method for obtaining PVC films by casting from a THF solution. Selected plasticizers were used to obtain PVC blends at 50 phr. They were then tested for plasticizer migration, hardness, thermogravimetric analysis, differential scanning calorimetry, and mechanical strength. Their properties were compared with the commercially available monomeric plasticizers di(2-ethylhexyl) terephthalate and di(2-ethylhexyl) adipate. The conducted study shows that the oligoesters obtained at a molar ratio of ADA to DFA of 9:1 and using an excess of 2-EH exhibit very good compatibility and plasticizing ability. The use of higher amounts of DFA worsens the compatibility of the oligoesters with PVC. However, a 4:1 ADA-to-DFA molar ratio produced results that still allow for the use of these compounds as plasticizers at lower concentrations or in combination with other plasticizers.

## 1. Introduction

Poly(vinyl chloride) (PVC) is one of the most widely used polymers in the world, after polyethylene and polypropylene. In 2023, PVC production was reported to be about 53 million tons [1]. PVC is used in a wide array of industries, ranging from building and construction, electrical and electronic equipment, healthcare (more than a quarter of polymer products in medicine are made of PVC), and the automotive industry down to mass-produced items such as packaging and clothing. It is a thermoplastic polymer, produced at an affordable cost in many different variants and with various properties: it is very durable, oil/chemical- and water-resistant, fire-retardant, and self-extinguishing. Approximately 60–70% of the annual production falls to rigid PVC, used primarily for window profiles and water pipes. The remainder 30–40% is utilized as ”soft” (also called flexible) PVC used for cable insulation, film, and flooring manufacturing [2,3,4]. However, PVC in its unaltered form is a hard and brittle polymer. This property stems from the polarity of the C–Cl bond and the strong dipole–dipole interactions, which limit the polymer chain mobility. Further difficulties in processing this polymer come from its degradation at elevated temperatures of 130 °C and above, alongside the release of hydrogen chloride. Because of that, adapting PVC to various processing techniques like extrusion, calendaring, molding, and coating requires mixing with different additives, which improve the processing properties of PVC by increasing the flow ability and protecting against the thermal decomposition under processing conditions [2,5].

**Figure 1:**
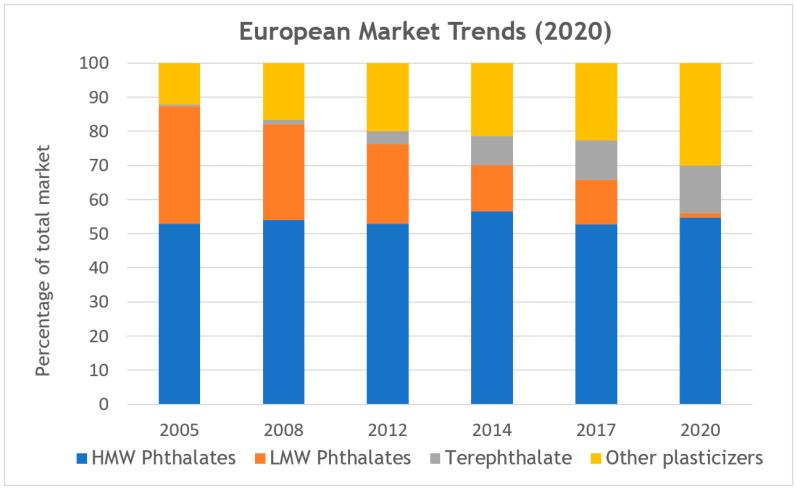
Trends in the European market share of various PVC plasticizer groups [3].

Plasticizers are the most common additives used in PVC, with the aim of lowering the material’s glass transition temperature (T_g_)—facilitating its processing. The plasticization of PVC allows us to obtain soft and flexible products, which are designated as PVC-P (plasticizer content up to 50–70%), and hard and rigid products, which are designated as PVC-U (plasticizer content up to 5%). Approximately 80–90% of the global consumption of plasticizers is dedicated to PVC processing. The esters of ortho-phthalic acid with linear or branched aliphatic alcohols of a long chain length (named ortho-phthalates or phthalates) are the most widely consumed plasticizers due to their good heat stability during processing, high degree of compatibility with the polymer, and price [3,6]. However, these compounds—especially low-molecular-weight (LMW) ortho-phthalates, including the most commonly used di(2-ethylhexyl) phthalate (DEHP)—easily migrate out of the polymer matrix with time. Numerous studies have demonstrated their potential to act as endocrine disruptors in humans and animals and the hazard they pose to the environment at large [7,8,9,10,11]. Due to those concerns, their use is currently subject to restrictions or prohibitions by the directives issued by the European Union (EU). In response to those regulations, the European plasticizer market has evolved significantly over the years, as shown in Figure 1. There has been a transition from LMW *ortho*-phthalates (e.g., diisobutyl phthalate (DIBP), dibutyl phthalate (DBP), benzyl butyl phthalate (BBP), and DEHP) (identified as “may cause cancer” under the REACH regulation) to higher-molecular-weight (HMW) phthalates. Unfortunately, outside the EU, LMW *ortho*-phthalates still account for about 35% of global consumption as they are widely produced and used in China, India, and other regions in Asia, the Middle East, Africa, and Latin America [3,6,9].

In response to the mounting regulatory pressure, there has been an increase in research focused on developing alternative plasticizers that are non-toxic, offer low migration, and are based on renewable raw materials whilst offering plasticization efficiency comparable to that of phthalates. Various methods have been proposed to obtain the most suitable plasticizer. One of them is the use of polymeric plasticizers, which offer lower volatility and greater durability compared to monomeric plasticizers but are more difficult to use and usually more expensive. Polymeric plasticizers are compounds that, in their structure, contain repeating molecular units [12]. Polymer plasticizers have a molecular weight in the range of 1000–10,000 according to the “*Handbook of Plasticizer*” by G. Wypych [6]. This group generally includes saturated polyesters [13], e.g., oligoisosorbide esters [14] and lactone-based aliphatic copolyesters [15]. One of the obvious candidates from an economic standpoint is vegetable oils, which are already the most widely used renewable raw materials for the chemical industries. However, unmodified vegetable oils are largely incompatible for use as plasticizers with PVC due to their low polarity. Various solutions to this problem have been described in the scientific literature and patents, such as epoxidized vegetable oils [16,17,18,19], epoxidized fatty acid esters [20,21], vegetable oil-based polyesters [22,23,24], and cardanol-based compounds [25,26].

In our research, we propose an innovative solution of using saturated dimerized fatty acid (DFA)—Pripol^®^ 1009 as a raw, renewable material to obtain new oligoester plasticizers for PVC. This approach helps prevent reactions involving unsaturated bonds or epoxy groups in the derivatives from vegetable oils during plasticization, which often leads to a significant increase in system viscosity and uneven gelation. Using sustainable, saturated dimerized fatty acids for PVC blends can offer more environmentally safe and sustainable products.

In addition, for the synthesis of new DFA-based oligoesters, triethylene glycol (TEG), adipic acid (ADA), and 2-ethylhexanol (2-EH) were used as raw materials to improve the compatibility of the resulting product with PVC. Selected plasticizers that passed the initial compatibility test using the solution casting method were used to produce PVC blends. The physicochemical, mechanical, and thermal properties were studied, which determine their plasticizing effect in PVC blends. In addition, a plasticizer migration study was performed from the PVC blend matrix. Solubility parameters for the plasticizer–PVC system were calculated to verify the system’s compatibility. Using sustainable saturated dimerized fatty acid for PVC blends can offer more environmentally safe and sustainable products.

## 2. Experimental Methods

### 2.1. Materials

The following chemicals were used as received: triethylene glycol (TEG), adipic acid (ADA) (Sigma-Aldrich, Merck, Steinheim, Germany), 2-ethylhexanol (2-EH) (Grupa Azoty ZAK S.A., Kędzierzyn-Koźle, Poland), petroleum ether (Pol-Aura, Avantor, Gliwice, Poland), and acetone (Pol-Aura, Chempur, Piekary Śląskie, Poland). Pripol^®^ 1009 from Cargill (formerly Croda, Mortara, Italy) was a mixture of 98.7% hydrogenated dimer fatty acids (DFA) and a maximum of 1% trimer fatty acids with 0.1% maximum fatty acids. The acid value of Pripol 1009 was 196 mg KOH/g.

Fascat 4100, i.e., poly(butyltin oxide)—used as a catalyst for transesterification and esterification reactions—was supplied from PMC Organometallix (Carrollton, TX, USA).

The plastic blends were prepared using PVC type S-70 (Anwil S.A., Włocławek, Poland), thermal stabilizer—Baeropan R 8890 KA/2 (Baerlocher, Unterschleissheim, Germany), with calcium carbonate used as a filler (Piotrowice Sp. z o.o., Zawichost, Poland). The PVC film samples, obtained by casting with solution, were produced with THF with 99.5% purity (Sigma-Aldrich, Merck, Steinheim, Germany). Low-density polyethylene (LDPE) sheets—LOTRENE^®^ FD 0474 (Qatar Petrochemical Company Ltd., Doha, Qatarwere used for the migration test. Di(2-ethylhexyl) terephthalate (DEHT) and di(2-ethylhexyl) adipate (DEHA) were used as primary plasticizers (Grupa Azoty ZAK S.A., Kędzierzyn-Koźle, Poland).

Chloroform-d (deuterated chloroform, CDCl3) containing 1% (*v*/*v*) tetramethylsilane (TMS), tetrahydrofuran (THF) containing 200–400 ppm butylated hydroxytoluene (BHT) as inhibitor, and a standardized solution of potassium hydroxide in 0.1 M ethanol (for titration) were used as supplied from Sigma-Aldrich (Merck, Steinheim, Germany).

### 2.2. Test Methods

**Nuclear Magnetic Resonance** (NMR) spectra were recorded using a Varian Unity INOVA 600 MHz spectrometer (Agilent Technologies, Santa Clara, CA, USA). NMR samples were prepared in deuterated chloroform. Tetramethylsilane (TMS) was used as an internal standard.

**Molecular weights and dispersities (Ð)** were determined by size exclusion chromatography analysis (SEC) in THF (including inhibitor BHT) suitable for HPLC as a solvent, with a flow rate of 1.0 mL/min at 40 °C, calibrated using linear polystyrene standards (162 to 56,600 g/mol). The SEC system (Agilent 1260 Infinity series, Agilent Technologies, Santa Clara, CA, USA) was equipped with a differential refractometer MDS RI Detector (Agilent Technologies, Santa Clara, CA, USA). A precolumn PLgel 3 μm Guard 50 × 7.5 mm, PLgel 3 μm MIXED E 300 × 7.5 mm column, and PLgel 5 μm MIXED C 300 × 7.5 mm column were used for separation.

**The acid value (AV)** determination was conducted according to the standard EN ISO 660 [27]. An amount of 0.5–1.5 g of the samples was taken and then dissolved in a mixture of 10 mL of acetone with 5 mL of petroleum ether and titrated with 0.1 M KOH in ethanol in the presence of phenolphthalein.

**Differential scanning calorimetry (DSC)** analyses were carried out using a DSC 3 Mettler-Toledo (Mettler Toledo, Greifensee, Switzerland). The samples of 8–10 mg were weighed in aluminum crucibles (40 μL). Measurements were carried out at a heating rate of 10 °C/min from −80 °C to 120 °C and cooled at the same rate in an atmosphere of nitrogen with a flow rate of 50 mL/min. The measurement for each sample was repeated twice. T_g_ was determined during the second heating run.

**Thermogravimetric analysis (TGA)**: The decomposition temperature of plasticizers and PVC plastics was determined by thermogravimetric analysis (TGA) using a Mettler Toledo 851e TGA/DTA analyzer (Mettler Toledo, Greifensee, Switzerland). The samples of 8–15 mg were weighed in alumina oxide crucibles (70 μL). Measurements were carried out under the following conditions: heating from 30 to 600 °C, with a heating rate of 20 °C/min, in a nitrogen atmosphere (flow rate of 50 mL/min).

**Measurement of plasticizer viscosity** was performed according to ISO 255 [28]. The dynamic viscosity of the plasticizers was tested on an Anton Paar SVM 3001 (Anton Paar, Graz, Austria) at 25 °C. This device is equipped with a precise system of configured coaxial cylinders, in which the inner cylinder rotates under electromagnetic force, and the resulting fluid resistance is recorded. Based on this, the system determines the dynamic viscosity value. The system also allows for determining the density based on the frequency of oscillation of the sample in the U-shaped vibrating tube. The sample was introduced via an autosampler, and the measurement temperature was stabilized with an accuracy of ±0.005 °C. The instrument automatically draws the amount of sample required for viscosity determination (typically 2.5 to 3.0 mL for a single measurement). The instrument performs at least two repetitions, and the maximum permissible deviation between the dynamic viscosity results should not exceed 0.35% of the average value.

**Color tests** of the liquid samples were conducted on a LANGE LICO 620 instrument (HACH, Berlin, Germany). Measurements were conducted with two ISO/ASTM color scales: the Hazen/APHA/Pt-Co scale and iodine scale.

**Mechanical properties** (tensile strength, elongation at break, Young’s modulus) of the PVC samples were evaluated using the universal testing machine (Zwick Roell 10kN Allround, Ulm, Germany, table-top universal testing machine) at room temperature. Mechanical test specimens were printed in the shape of a dumbbell (type 1BA) using a pneumatic press (Zwick Roell, series 7108, Ulm, Germany), according to SIST EN ISO 527-2 [29]. The specimen bars were tested at a strain rate of 50 mmˑmin^− 1^ with a 10,000 N load cell (for extruded plastic) and with a 100 N load cell (for solvent-casted films). All values are reported as the average of at least five samples.

**A hardness** test was carried out according to PN-EN ISO 868 [30] on a Zwick/Roell 3105 combi test apparatus (Zwick Roell, Ulm, Germany) in the Shore A range at 15 s. Two sheets of pressed PVC, each 2 mm thick, were joined together. The hardness test was conducted at five different locations of the joined sheets, and the average of the five measurements is given as the result.

**A migration test** of the plasticizer from the sample plasticized PVC was carried out as per EN ISO 177 [31]. This measurement determines the weight loss with a 50-millimeter-diameter disc of a sample PVC when in contact with rigid LDPE sheets, at 70 °C, under a stress of 5 kg. Weight measurements were taken independently at different intervals: 2, 4, 7, and 28 days. The percentage loss of plasticizer from PVC material was calculated according to the following formula:

(1) $$\%\;loss\;of\;plasticizers=\frac{{(w_{s}-w}_{e})\cdot 100\%}{w_{s}\cdot \frac{50}{164.5}}$$

where

*w_s_*—“starting weight”—the weight of the disc before the start of the migration test;

*w_e_*—“ending weight”—the weight of the disc after the migration test at the selected time;

50—parts per hundred resin (phr) of the plasticizer used to create the dry blend;

164.5—parts phr of all ingredients used to create the dry blend, i.e., PVC, plasticizer, chalk, and thermal stabilizer.

All values are reported as the average of at least five samples.

### 2.3. Synthesis of Plasticizers

New plasticizers were obtained in two-step synthesis using the one-pot method. In the first step, an oligoester was obtained from adipic acid (ADA), dimeric fatty acid (DFA)—Pripol 1009^®^, and triethylene glycol (TEG) in the appropriate molar ratios (Table 1). Fascat 4100 was used as the catalyst. The molar ratio of (ADA + DFA):TEG in these types of syntheses was constant 1.06:1, while the molar ratio of DFA:ADA was 1:9 or 1:4. Substrates were put into a three-necked glass flask equipped with a Dean–Stark trap, reflux condenser, a magnetic stirrer, a gas inlet (nitrogen), and a thermometer connected to the temperature regulator of the heating mantle. The temperature of the reaction was between 170 and 180 °C. The increase in viscosity of the mixture and stabilization of the AV at a constant level, around 30–40 mg KOH/g, indicates an overreaction of the glycol and marks the end of the first step of synthesis. In the second step, the next portion of the Fascat 4100 catalyst and 2-EH were introduced in situ into the reaction system in an amount such that the molar ratio of hydroxyl groups of the reactants to carboxyl groups of the reactants (x) was 1.10, 1.15, or 1.35, depending on the assumptions.

(2) $$\frac{{2\cdot n}_{TEG}+n_{2-EH}}{2{\cdot n}_{DFA}+2{\cdot n}_{ADA}}=\frac{n_{OH}}{n_{COOH}}=x$$

This is the formula for the molar ratio of hydroxyl groups (-OH) to carboxyl groups (-COOH), where

n*_TEG_*—the number of moles of triethylene glycol;

n*_2-EH_*—the number of moles of 2-ethylhexanol;

n*_DFA_*—the number of moles of dimerized fatty acid, Pripol 1009^®^;

n*_ADA_*—the number of moles of adipic acid;

n*_OH_*—the number of moles of hydroxyl groups of the reactants;

n*_COOH_*—the number of moles of carboxyl groups of the reactants.

The synthesis was complete when the AV of the obtained product reached approximately 1 mg KOH/g. Subsequently, the excess 2-EH was distilled off at T = 150–160 °C under *p* < 50 mbar. The obtained product was homogeneous, clear, and stable during storage. The general formula of oligoester can be represented as follows:

2EH − [ADA − TEG]_n_ − [DFA − TEG]_m_ − ADA − 2EH (3)

Compounds containing only one dicarboxylic acid were synthesized as reference plasticizers: ADA (PD_13, Table 1) or DFA (PD_03, Table 1).

To verify the importance of the timing of introducing DFA into the reaction system, a three-step synthesis was also carried out. After polycondensation of ADA with TEG to an AV corresponding to a trimer of these substrates of about 70 mgKOH/g, DFA was added in the second step with another portion of the catalyst. After reaching an AV of about 30 mgKOH/g, 2-EH was added in excess in the third step of the process. The synthesis was carried out until an AV of about 1 mg KOH/g was reached.

Oligoester based on DFA, TEG, and 2-EH (PD_03):

**^1^H NMR** (CDCl_3_, 600 MHz) δ [ppm]: 0.78–0.94 (C**H_3_**- from DFA units and 2-EH units); 1.12–1.43 (-C**H_2_**- from DFA units and 2-EH units); 1.51–1.67 (-C**H**< from DFA units and 2-EH units); 2.25–2.37 (-C**H_2_**-COO- from DFA units); 3.59–3.78 (-CH_2_-O- from TEG units); 3.94–4.02 (-C**H_2_**-OOC- from 2-EH units); 4.20–4.27 (-C**H_2_**-OOC- from TEG units);

**^13^C NMR** (CDCl_3_, 150 MHz) *δ* [ppm]: 11.12 (**C**H_3_- from 2-EH units); 14.10–14.42 (**C**H_3_- from 2-EH and DFA units); 22.81, 25.04, and 25.18 (-**C**H_2_- from DFA units); 23.09, 23.93, 29.05, and 30.56 (-**C**H_2_- from 2-EH units); 29.14–30.50 (-**C**H_2_- from DFA units); 32.05 (-**C**H< from DFA units); 34.32 and 34.58 (-**C**H_2_-COO- from DFA units); 38.88 (-**C**H< from 2-EH units); 61.83 and 61.88 (-**C**H_2_OH from TEG units); 63.30 and 63.41 (-**C**H_2_OOC- from TEG units); 66.74 (-**C**H_2_OOC- from 2-EH units); 69.34, 69.38, 70.47, and 70.67 (-**C**H_2_O- from TEG units); 72.58 (-O**C**H_2_CH_2_OH from TEG units); 173.91 and 173.97 (-**C**OO- TEG with ADA); 174.21 (-**C**OO- 2-EH with ADA).

Oligoester based on ADA, TEG, and 2-EH (PD_13):

**^1^H NMR** (CDCl_3_, 600 MHz) *δ* [ppm]: 0.86–0.94 (C**H_3_**- from 2-EH units); 1.23–1.40 (-C**H_2_**- from 2-EH units); 1.51–1.60 (-C**H**< from 2-EH units); 1.62–1.72 (-C**H**_2_- from ADA units); 2.30–2.42 (-C**H_2_**-COO- from ADA units); 3.59–3.78 (-C**H_2_**-O- from TEG units); 3.94–4.02 (-C**H_2_**-OOC- from 2-EH units); 4.20–4.27 (-C**H_2_**-OOC- from TEG units);

**^13^C NMR** (CDCl_3_, 150 MHz) *δ* [ppm]: 10.99 and 14.04 (**C**H_3_- from 2-EH units); 22.95, 23.78, 28.90, and 30.40 (-**C**H_2_- from 2-EH units); 23.97–25.06 (-**C**H_2_- from ADA units); 33.00–35.00 (-**C**H_2_-COO- from ADA units); 38.72 (-**C**H< from 2-EH units); 61.69 (-**C**H_2_OH from TEG units); 63.00–63.80 (-**C**H_2_OOC- from TEG units); 66.79 (-**C**H_2_OOC- from 2-EH units); 68.50–69.70 and 69.90–71.00 (-**C**H_2_O- from TEG units); 72.50 (-O**C**H_2_CH_2_OH from TEG units); 173.24 (-**C**OO- TEG with ADA); 173.51 (-**C**OO- 2-EH with ADA).

Plasticizer based on DFA, TEG, ADA, and 2-EH:

**^1^H NMR** (CDCl_3_, 600 MHz) δ [ppm]: 0.78–0.96 (C**H_3_**- from 2-EH units and DFA units); 1.13–1.45 (-C**H_2_**- from 2-EH units and DFA units); 1.51–1.72 (-C**H**< from 2-EH units and DFA units; -C**H_2_**- from ADA units); 2.25–2.43 (-C**H_2_**-COO- from DFA units and ADA units); 3.60–3.78 (-C**H_2_**-O- from TEG units); 3.94–4.02 (-C**H_2_**-OOC- from 2-EH units); 4.20–4.30 (-C**H_2_**-OOC- from TEG units);

**^13^C NMR** (CDCl_3_, 150 MHz) *δ* [ppm]: 11.10 (**C**H_3_- from 2-EH units); 14.07–14.39 (**C**H_3_- from 2-EH and DFA units); 22.81, 25.04, and 25.11 (-**C**H_2_- from DFA units); 23.08, 23.90, 29.03, and 30.52 (-**C**H_2_- from 2-EH units); 24.18–24.76 (-**C**H_2_- from ADA units); 29.17–30.57 (-**C**H_2_- from DFA units); 32.03 (-**C**H< from DFA units); 33.61–34.88 (-**C**H_2_-COO- from ADA units and from DFA units); 38.84 (-**C**H< from 2-EH units); 61.87 (-**C**H_2_OH from TEG units); 63.21–63.86 (-**C**H_2_OOC- from TEG units); 66.93 (-**C**H_2_OOC- from 2-EH units); 69.10–69.63 and 70.14–71.08 (-**C**H_2_O- from TEG units); 72.57 (-O**C**H_2_CH_2_OH from TEG units); 173.25–173.52 (-**C**OO- TEG with ADA); 173.60 (-**C**OO- 2-EH with ADA); 173.92 (-**C**OO- TEG with DFA), 174.21 (-**C**OO- 2-EH with DFA).

### 2.4. Preparation of PVC Film Casting Method—The Compatibility Test

The PVC film casting method described in the literature was used to preliminarily determine the compatibility of suspension PVC with the synthesized plasticizers. This method is faster and does not require as many raw materials as in the conventional method, where you create moldings from granules obtained from a dry blend on a twin-screw extruder [32,33], therefore, it was suitable for preliminary testing.

Solution-cast PVC films were prepared by dissolving 7.66 g of PVC type S-70 and 3.83 g (50 phr) of plasticizer in 100 mL of THF. The solution was stirred using a magnetic stirrer (400 rpm) at 50 °C, with a reflux condenser, until a homogeneous, liquid mixture was obtained (3–4 h). The prepared solutions were cast into a Petri dish with a diameter of ø 18 cm, covered with a lid, and dried at room temperature under atmospheric pressure to remove most of the THF. After approximately 48–72 h, solid films were removed from the dish and placed in a vacuum dryer at 40 °C for 24 h to completely evaporate the THF. Thin films with a thickness of approximately 0.5 mm were obtained. The PVC films were conditioned in a desiccator for several days, and then their transparency, flexibility, and plasticizer migration (indicated by a greasy film surface) were visually and tactually checked. The T_g_ of the product was determined using DSC. To provide context for the results, similar films were obtained using DEHT as a commercial plasticizer and without any plasticizer. 

### 2.5. Preparation of PVC Blends

To prepare all PVC dry blends, a mixture with the same composition was used as follows: PVC type S-70—100 phr, plasticizers—50 phr (total), calcium carbonate (CaCO_3_)—10 phr, thermal stabilizer—4.5 phr. A High-Power Jacketed High-Speed Laboratory Mixer from LabTech Engineering Company LTD was utilized to prepare PVC dry blends. The polymer and thermal stabilizer were initially mixed at 2500 rpm until the blends’ temperature reached 80 °C. Subsequently, the mixing speed was reduced to 1800 rpm, and the plasticizer was introduced. At 95 °C, calcium carbonate was incorporated into the blend, which was then mixed until it reached a temperature of 110 °C. Following this, the blend was transferred into a cooling bowl and cooled with water at 750 rpm for 10 min. This entire process took approximately 25 min. The dry blend was then allowed to season at room temperature for 24 h.

For extrusion, an intermeshing twin-screw extruder (ZAMAK Mercator, Skawina, Poland, screw diameter: 24 mm, screw length: 40 D) equipped with a cooling bath and a pelletizer was employed. The extruder, featuring nine heating zones, operated with screw rotation at 35 min^−1^ and a temperature range from 40 °C (hopper) to 160 °C (die head). Pellets were subsequently compressed using a benchtop hydraulic press (Labtech, Labtech Engineering Co., Thailand, The Micro Scientific Bench Top Hydraulic Presses Type LP30-B) to produce sheets of about 2 mm thickness. This compression was performed at 180 °C, 16 MPa for 3.5 min, followed by 4 min of cooling. The prepared extrudate was stabilized in a conditioning chamber at a temperature of 25 °C and relative humidity of 50% for 24 h, after which the properties of the finished products were analyzed.

### 2.6. Statistical Analysis

For statistical analysis, MS Office ver. 2.5.0 and MS Excel 2010 were used. The obtained mechanical property resultswere subjected to descriptive statistics. The results shown in the charts represent the average values from a minimum of 5 measurements. The standard deviation (σ) of the obtained result is presented on each chart in the form of “error bars”. For each sample tested, the standard deviation criterion—the 3-sigma rule—was applied, according to which, if a result deviates from the mean by more than 3 standard deviations (3σ), it is considered an outlier and excluded from the analysis.

## 3. Results and Discussion

### 3.1. Synthesis of Plasticizers and Their Compatibility Test with PVC

**Figure 2:**
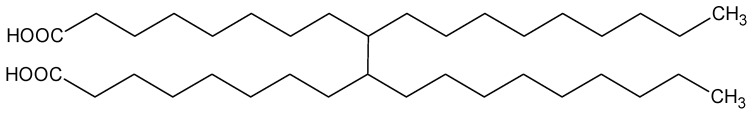
Structural formula of the DFA molecule accounting for the largest percentage in Pripol^®^ 1009.

The new oligomeric plasticizers based on saturated dimer fatty acid (DFA), adipic acid (ADA), triethylene glycol (TEG), and 2-ethylhexanol (2-EH) were obtained by synthesis of a two-step, one-pot method. Pripol^®^ 1009 was used as the DFA. Pripol™ 1009 F is a biobased, distilled, and high-purity hydrogenated DFA in 98.7%. It contains 100% renewable carbon. Qualitative analysis of this compound, conducted using the LC qToF technique, proved that the main components are molecules with a molar mass of 566 g/mol and a molecular formula of C_36_H_70_O_4_, as well as molecules with a molar mass of 564 g/mol and a molecular formula of C_36_H_68_O_4_. Figure 2 shows a hypothetical structural formula of Pripol 1009, which is likely the major component in the mixture of DFA. The main advantages of this compound, reported by the manufacturer, are its flexibility, thermo-oxidative stability, and chemical resistance. Furthermore, the monomer exhibits hydrolytic stability and good pigment-wetting properties.

In the case of synthesizing plasticizers using vegetable oils, unsaturated bonds are often subjected to an epoxidation process, which constitutes an additional step. On an industrial scale, the epoxidation of vegetable oils is carried out using peroxy acids in the presence of mineral acids as catalysts. This method is characterized by high energy consumption and the production of significant amounts of wastewater that is difficult to treat. Additionally, it requires careful temperature and process-time monitoring, presents problems in separating the catalyst from the reaction products, and is known to degrade the synthesis apparatus by corrosion [16,34]. Oxirane groups in epoxidized vegetable oils increase the compound’s polarity and improve compatibility with PVC but compatibility is still so low that they are used at best as secondary plasticizers. Commercially available epoxidized soybean oil (ESO) is used mainly as a heat stabilizer and only to a limited extent as a secondary plasticizer in the processing and production of PVC products. In addition, epoxy compounds are less stable by their reactivity, which, as mentioned earlier, often leads to a significant increase in system viscosity and uneven gelation [16,17,18,20,21,35,36]. In our studies, it was decided to increase the polarity of the plasticizer by using a glycol with the appropriate number of ether groups—TEG. According to the literature data, the more ether groups there are in the glycol molecule, the better the plasticizer’s compatibility with PVC [26,33,37]. In addition, TEG has a high boiling point of 285 °C and, thus, relatively low vapor pressure at the process’s temperature (180 °C), which means minimal loss during processing due to evaporation and condensing with water into the Dean–Stark trap.

Our research began with the synthesis of plasticizers using one dicarboxylic acid, DFA (PD_03) or ADA (PD_13), in reaction with TEG and 2-EH. These products were used to verify their compatibility with PVC and as reference plasticizers.

All reactions were carried out at 170–180 °C, using 0.2 wt% butylstannoic acid (Fascat 4100) as a catalyst based on the results in earlier plasticizer studies involving vegetable oils [21]. In each synthesis, the number of moles of the acid or acids used in the first step of the reaction was higher than the number of moles of TEG. However, the total number of moles of hydroxyl groups from TEG and 2-EH in the process was used more than the total number of carboxyl groups to make a product with an AV < 1 mgKOH/g. An excess of 2-EH relative to the number of moles of carboxyl groups guarantees that the carboxyl groups are completely converted and a non-reactive product is obtained.

Table 2 presents selected properties of the obtained plasticizers, such as average molecular weight ${\bar{(M}}_{n}$ and ${\bar{M}}_{w}$), dispersion index (Ð), viscosity, density, and color. This allows for a preliminary comparison of the products with each other.

**Figure 3:**
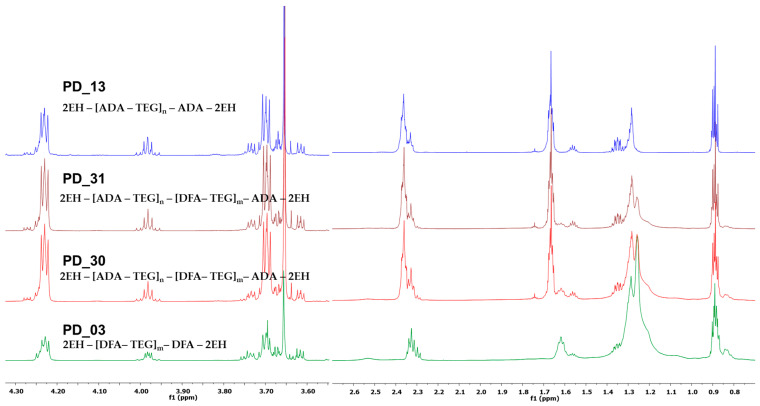
Comparison of fragments of ^1^H NMR (CDCl_3_, 600 MHz) spectra of plasticizer based on DFA and ADA (PD_30 and PD_31), plasticizer based on DFA (PD_03), and plasticizer based on ADA (PD_13).

**Figure 4:**
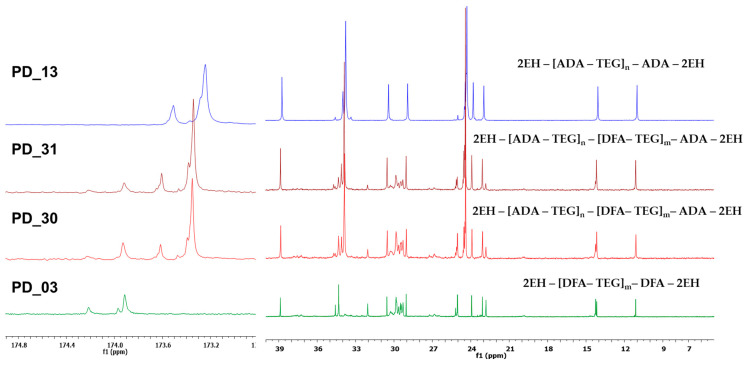
Comparison of fragments of ^13^C NMR (CDCl_3_, 150 MHz) spectra of plasticizer based on DFA and ADA (PD_30 and PD_31), plasticizer based on DFA (PD_03), and plasticizer based on ADA (PD_13).

The obtained plasticizer of the PD_03 (only DFA) was clear, yellow (440 Hazen scale), and permanently stable. In comparison, the product PD_13 (only ADA) was clear and colorless for about two years, after which a small amount of precipitate appeared. The main difference between these plasticizers was their viscosity. The viscosity of PD_13 was 380 mPa·s, whereas PD_03 had a significantly higher viscosity of 2860 mPa·s. The obtaining of oligomeric compounds was confirmed by SEC analysis, in which the average M_w_ of the products was determined—4720 Da for PD_03 and 2490 Da for PD_13. On the other hand, the obtaining of oligoester compounds is indicated by characteristic signals of protons of -CH_2_-COO- groups from ADA or DFA units in the range of 2.25–2.42 ppm, protons of -CH_2_OOC- groups from 2-EH units in the range of 3.94–4.02 ppm, and from TEG units in the range of 4.20–4.27 ppm present on ^1^H NMR spectra (Figure 3). In addition, characteristic signals of carbon atoms of ester groups are present on the ^13^C NMR spectra at 173.5 ppm bonding ADA units with 2-EH and 173.2 ppm bonding ADA units with TEG for sample PD 13 (blue line, Figure 4) and 174.2 ppm bonding DFA units with 2-EH and 173.9 ppm bonding DFA units with TEG for sample PD_03 (green line, Figure 4).

In the next step, to preliminarily determine the compatibility of PVC in the system with the obtained compounds, a method was used to obtain PVC films by casting from the THF solution. This method allowed us to pre-select the most promising plasticizers in a more cost- and time-efficient manner when compared to creating moldings from pellets obtained from a dry blend on a twin-screw extruder. Plasticizers whose PVC films showed similar properties to DEHT films, and thus showed the best promise for industrial use, were selected for the next testing step—the PVC dry-blend preparation.

Unfortunately, the plasticizer based on only one acid—DFA (PD_03)—was found to be incompatible with PVC. The film was inhomogeneous; after THF evaporation at room temperature, it had white discoloration—probably parts of the surface where the plasticizer was not present. During drying under vacuum, the surface of the film became greasy and rigid. Thus, in a relatively short time, the film achieved properties similar to unplasticized film, which was also white and rigid. This was also confirmed by the DSC analysis, in which the T_g_ of the formed film was determined to be 79 °C for the PVC film with PD_03—this result is very close to that of the unplasticized PVC sample at 80 °C (Table 3).

The compatibility between a plasticizer and a polymer is influenced by parameters such as polarity, chemical groups, chain length, number of hydroxyl groups, number of ester bonds, molecular weight, and so on. To achieve a high degree of compatibility, the plasticizer’s polarity and the polymer’s polarity must be at a similar level. However, the molecules of DFA have very limited polarity because of their long aliphatic chains. It is safe to say that using only this acid in the polycondensation reaction did not obtain a good PVC plasticizer. According to the literature, PVC with aliphatic (poly/oligo)esters is compatible mainly through the formation of hydrogen bonds between the carbonyl group of the ester and the hydrogen atom of the >C(H)Cl group of the polymer repeating units. In addition, based on the studies reported in publications, it was observed that the ratio of -CH_2_- to >C=O groups in the (poly/oligo)ester is significant, and the best results are obtained when it is between 4 and 10 [35,38,39].

Table 3 presents a summary of the preliminary assessment of the general properties of PVC films obtained using the selected plasticizers. If the film exhibited the specified characteristics (transparency, flexibility, and dry surface), it was marked with a “+” symbol. Otherwise, a “−” symbol was assigned. Only plasticizers for which the films received three “+” marks in the summary were selected for further investigation.

When a film was made using PD_13 plasticizer (ADA only), a partly opaque material was obtained after THF evaporation at room temperature, with physical shrinkage of about 20%. After it had been stored for some time, already dried under vacuum, the film became increasingly opaque and less flexible. However, this plasticizer was more compatible with PVC than PD_03, as indicated by the T_g_ of the resulting film of −25 °C.

**Figure 5:**
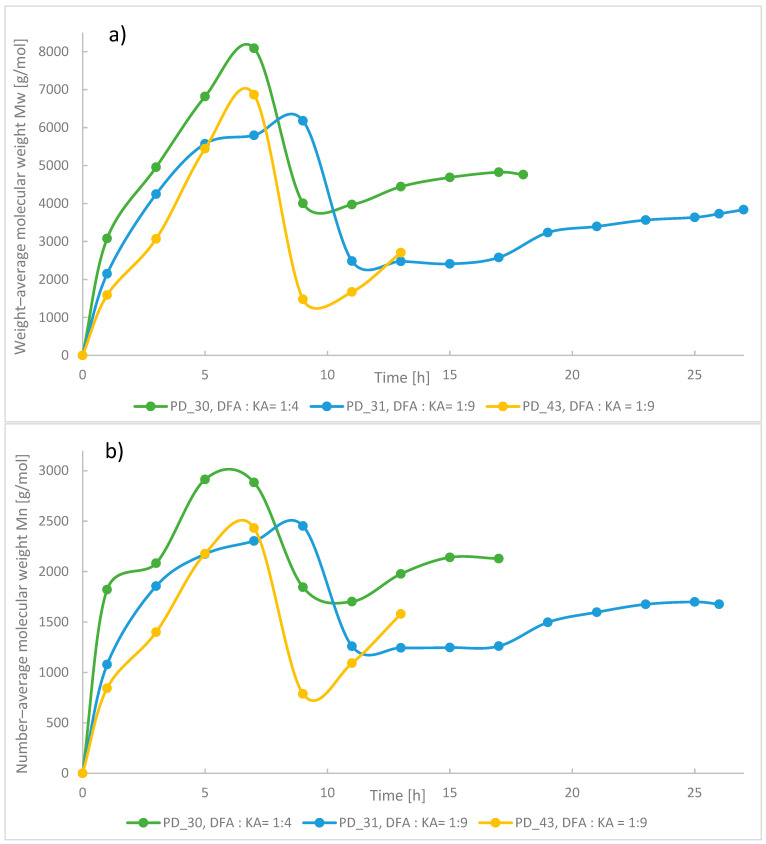
The change in the average M_w_ (**a**) and in the average M_n_ (**b**) values as a function of time for the selected syntheses.

Therefore, subsequent syntheses combined both acids at selected molar ratios (1:9 or 1:4) with TEG and the catalyst. At the end of the first step, the reaction system had a relatively high viscosity, and the obtained products had higher average molecular weights (the maximum value average M_w_ on the chromatogram, Figure 5a, compared to the final average M_w_ of the obtained plasticizers and Figure 5b compared to the final average M_n_).

**Figure 6:**
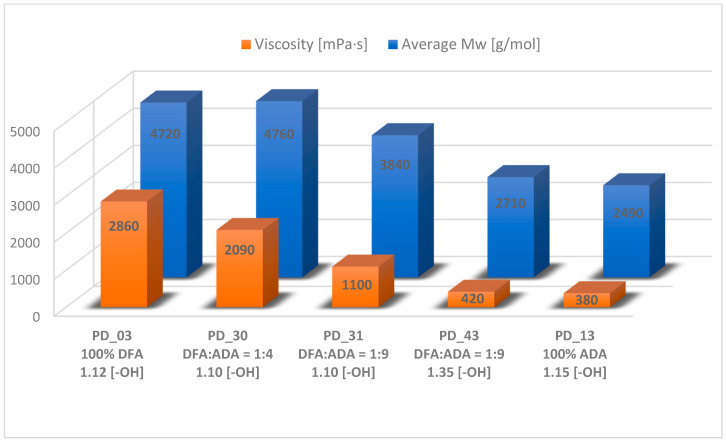
The viscosity (at 25 °C) and average M_w_ of the obtained plasticizers.

The first step was considered to be complete either when the AV value had stabilized—based on two subsequent samples taken 2 h apart (a difference of no more than 3–5 mgKOH/g)—or when the AV value had reached a level of 30 mgKOH/g. The second step was initiated by the addition of 2-EH in situ to the reaction system. 2-EH was introduced in such amounts that the molar ratio of the hydroxyl groups of the reactants, 2-EH and TEG, to the carboxyl groups of the reactants, ADA and FDA, was 1.10 or 1.35. The introduction of this alcohol into the system drastically reduced the viscosity of the system by dilution, which is observed in Figure 5a by a rapid decrease in the average M_w_. After that, the average M_w_ again increases slightly due to the condensation of the 2-EH molecules with carboxylic groups, which terminate macromolecule chains with 2-ethylhexyl groups. The amount of 2-EH had a fundamental effect on the average M_w_ and viscosity of the resulting plasticizer. Figure 6 compares these two properties in the products. Sample PD_43, which used much more 2-EH in its synthesis than the analogous PD_31—using the same amount of other raw materials (including DFA)—has a significantly lower viscosity and average M_w_. The reduction in the average M_w_ by about 1000 Da with a comparable dispersity index of the products was likely a result of the transesterification reaction of oligoester chains by 2-EH during distillation under vacuum. Moreover, a higher amount of 2-EH leads to a faster esterification reaction of free acid groups and the achievement of an AV below 1 mg KOH/g, which was the criterion for completing this synthesis. In addition, if more DFA was used in the synthesis (DFA:ADA = 1:4) with the same amount of 2-EH, then a plasticizer with a higher average M_w_ was obtained (PD_30 vs. PD_31). The 2-ethylhexyl part of the plasticizer chain improves its solubility and well-influences the plasticization process [38]. The use of ADA allowed for the production of a stable product, which, after initial PVC plasticization attempts using the solution casting method, showed very good plasticizing properties across a wide range of molar ratios of reagents.

The ^1^H and ^13^C NMR analyses also confirm that oligoester plasticizers based on DFA and ADA were obtained. Figure 3 shows a comparison of the ^1^H NMR spectra of oligoester samples consisting of DFA, ADA, TEG, and 2-EH (PD_30—1:4 = DFA:ADA and PD_31—1:9 = DFA:ADA) and reference oligoester samples PD_03 and PD_13. Characteristic signals for ester compounds were detected as follows: protons -CH_2_-COO- in the range of 2.25–2.43 ppm and protons -CH_2_OOC- in the range of 3.94–4.30 ppm. In addition, the signals of methyl groups in the range of 0.80–0.85 ppm and methylene groups in the range of 1.59–1.67 ppm of the aliphatic chain of DFA are present in the spectra of samples PD_30 and PD_31, which do not overlap with the signals of the 2-EH units (Figure 4) and confirm the presence of DFA units in the obtained plasticizer. On the other hand, in Figure 4—showing the comparison of ^13^C NMR spectra—clear evidence for the formation of oligoesters comprises the presence of carbon atoms of ester groups, where four signals for -COO- groups are visible for the reaction products DFA, ADA, TEG, and 2-EH in the following ranges: 173.25–173.52 ppm TEG with ADA;173.60 ppm 2-EH with ADA; 173.92 ppm TEG with DFA; 174.21 ppm 2-EH with DFA. In addition, the presence of a characteristic group of methylene signals in the range of 24.18–24.76 ppm—which have a higher intensity in PD 30 (DFA:ADA = 1:4) than in PD 31 (DFA:ADA = 1:9)—confirms the building of the units in DFA into an oligoester. Similarly, the presence of high signals of methylene groups in the ranges of 24.18–24.76 ppm and 33.61–34.21 ppm confirms the building of ADA units into the structure of the obtained product.

A three-step reaction was also performed to verify the effect of the stage at which DFA is introduced into the reaction system. The synthesis PD_14 began with the reaction of ADA with TEG at a temperature of 190 °C, using a 1.05-fold excess of glycol. After reaching an AV trimer of 70 mgKOH/g, the DFA was added. The second step was carried out until the AV decreased slightly and stabilized with 2–3 mg KOH/g between consecutive samples. Then, in the third step, 2-EH was introduced and finished the process when the AV was around 1 mgKOH/g. Unexpectedly, after about 2–3 months of storage, a precipitate began to separate from the product.

All synthesized plasticizer samples were made into films analyzed in a compatibility test. The results of this preliminary test aided the selection of plasticizers with the most promising properties for the next test phase, which was the preparation of PVC blends. The primary criteria for qualifying for further testing were the sample transparency and flexibility of the samples and the non-migration of the plasticizer to the surface, indicated by three pluses in Table 3. The T_g_ of the suitable samples were similar and in the −27 °C—−30 °C range. However, sample PD_14 was rejected due to the precipitate that formed after several months. Samples PD_17 and PD 43 essentially differed only in scale. Based on the above criteria, PVC blends were prepared using the PD_30, PD_31, and PD_43 plasticizers.

### 3.2. Characteristics of Plasticized PVC Blends

PVC dry blends were prepared with selected plasticizers in the amount of 50 phr in addition to calcium carbonate—10 phr and thermal stabilizer Ca/Zn—4.5 phr. Subsequently, the dry blends were processed into pellets using a twin-screw extruder. The pellets were subsequently pressed into sheets about 2 mm in thickness using a hydraulic press. The PVC blend preparation procedure was consistent for all samples, using the same quantitative and qualitative ingredients, adhering to the same conditioning times, and using the same testing method. This allows us to compare the properties of the finished products and be reasonably certain that the differences stem from the use of different plasticizers. Analyses of the thermal, mechanical, and physicochemical properties of molded samples cut from sheets of PVC blends were performed and compared with blends containing the commercial plasticizer DEHT or bis(2-ethylhexyl) adipate DEHA.

(a) Thermal properties

The thermal characteristics of plasticized PVC blends were determined through thermogravimetric analysis (TGA) from 30 to 600 °C and differential scanning calorimetry (DSC) from −80 °C to 120 °C using a cycling method of heating–cooling–heating with a ramp rate of 10 °C/min. Table 4 presents the T_g_ values for the PVC blends obtained with the selected oligoesters based on DFA and commercial plasticizers: DEHT and DEHA. In addition, the temperature at which there were losses of 5% in mass (the start of decomposition) and the temperatures of two stages of the decomposition were determined and recorded.

The main function of any plasticizer added to PVC is to lower its T_g_, which for neat PVC polymer is about 80 °C (Table 3—PVC film). PVC material below the T_g_ temperature becomes hard and brittle, often rendering it unusable. The same material above its T_g_ becomes more flexible. T_g_ values of −25 °C and −27 °C obtained in the blends of PVC with the new plasticizers PD_31 and PD_43 indicate their plasticizing effect on PVC. It can be assumed that these compounds diminish the intermolecular interactions among PVC chains and increase the free volume within the PVC network. The T_g_ results for the PVC with novel plasticizers are consistent between the initial cast films and the secondary dry-blend sample tests. In the case of the PVC sheet from PD_30, the temperature T_g_ could not be determined because its surface was greasy, which contributed to the difficulty in its determination. The explanation for this effect has been discussed in more detail later in the discussion of the results.

The decomposition of the PVC blends occurs in two mass-loss stages. In the first stage of decomposition, there is the most significant weight loss of the sample, which was about 80 wt% for DEHA and DEHT at 283 °C and 286 °C, respectively; 75 wt% for PD_43 and PD_31 and 55 wt% for PD_30 at 300 °C. At these temperatures, the elimination of HCl from the PVC primarily occurs and creates sequences of polyenes. At the same time, the plasticizer—especially low-molecular-weight plasticizers—can evaporate. The higher value of the first decomposition temperature for blends with DFA-based plasticizers can be explained by the thermostabilizing properties of DFA units and the low volatility of these compounds compared with DEHT and DEHA. During the second stage, in the 400–550 °C range, the polymer backbone breaks down into mainly aromatic and aliphatic hydrocarbons [40,41].

(b) Mechanical properties

**Figure 7:**
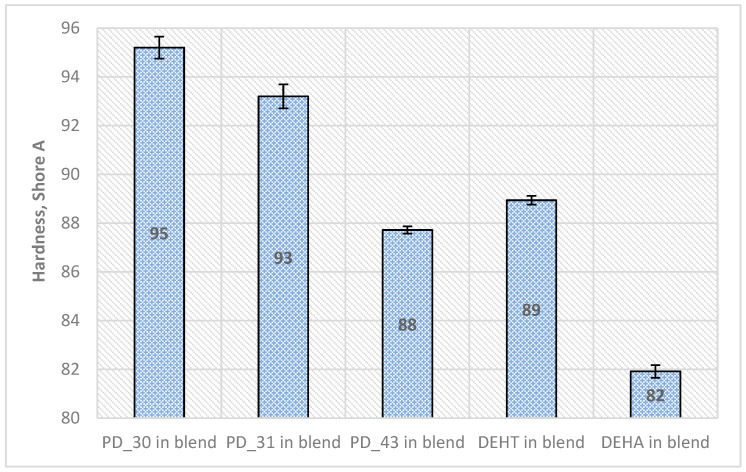
Shore A hardness of PVC blends (mean ± σ, n = 5).

The Shore hardness tester measures the resistance to indentation of various polymer types. The Shore A scale is preferred for ”softer” plastics, like polyolefins or other polyvinyls. The harder the material, the higher the value obtained in the test, with the Shore A scale ranging from 0 to 100 points. The hardness result of PVC is influenced, among other factors, by the content of the plasticizer. Due to the plasticizer’s migration, the hardness value changes over time. The obtained hardness result is one of the parameters of the mechanical strength of the product; however, this cannot be used to predict its other properties [42]. Figure 7 shows the hardness results of plasticized PVC materials with selected DFA-based plasticizers and comparatively with DEHT or DEHA. The obtained materials with DFA-based plasticizers have a hardness above 85 ShA, which classifies them as hard. The samples with the PD_43 plasticizer showed the lowest hardness of 88 ShA, about a similar hardness to the samples of PVC blends with DEHT—89 ShA. This is probably influenced by its average molecular weight (M_w_ = 2490 Da) and viscosity (η = 420 mPa·s), which were the lowest compared to PD_30 and PD_31. Only the DEHA plasticized PVC sample has a slightly lower hardness of about 82 ShA.

**Figure 8:**
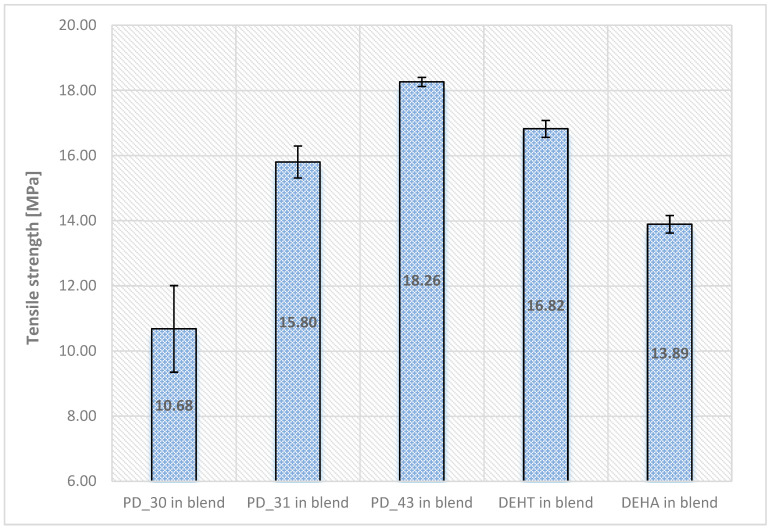
The tensile strength of plasticized PVC blends with new plasticizers (mean ± σ, n = 5).

**Figure 9:**
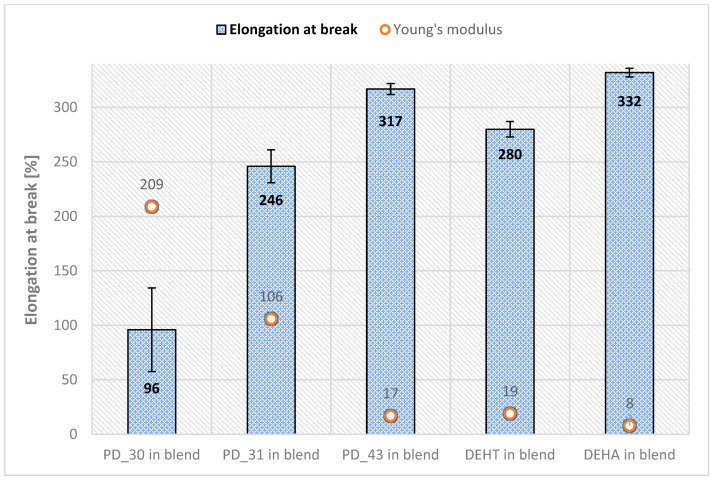
The elongation at break and Young’s modulus of plasticized PVC materials with new plasticizers (mean ± σ, n = 5).

In addition, a tensile test of samples was performed, and the results of tensile strength, elongation at break, and Young’s modulus, along with standard deviations, are shown in Figure 8 and Figure 9.

The presented results of the mechanical properties of the obtained new plasticizers show a certain trend of changes, depending on their viscosity, which is related to the proportion of 2-EH and DFA units in the average molecular weight of the plasticizer. The smaller the proportion of 2-EH and the larger the DFA, the less flexible the PVC blends were, meaning they were more rigid, as seen for PD_30 in the PVC blend. The best results were obtained for sample PD_43, which had the lowest Young’s modulus of 17 MPa, an elongation to break of 317%, and a relatively high breaking strength of 18.3 MPa. Lower values of Young’s modulus indicate that the plasticizer used to prepare a given sample shows higher plasticization efficiency and material elasticity compared to others, which is evident when compared with commercial plasticizers—DEHA and DEHT—whose values are in the range of 8–19 MPa.

It is important to note that the sample with the highest DFA content—PD_30 in the PVC blend—shows the largest standard deviation among the results. Such a result is not a coincidental one. All molds were produced using the same method and sample conditioning times. After extrusion, the pellets were left for 24 h before pressing. The properties of all molds were tested about 48 h after pressing. In the case of the PD_30 in the PVC blend, the surface became greasy after this time, lowering the results’ repeatability. The appearance of greasy spots on the surface indicates the poorer compatibility of this plasticizer with S-PVC. The greasy layer, which migrates to the surface of the PVC blend, was investigated with the use of ^1^H NMR and SEC. It was confirmed that the compound was the introduced plasticizer PD 30. The average Mw of the analyzed compound was also 4760 Da. The next section will present data to explain this observed phenomenon.

Migration

**Figure 10:**
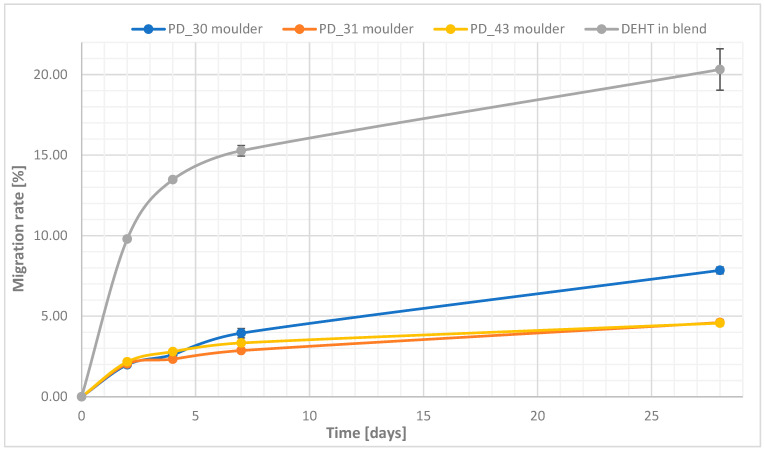
The migration of plasticizer with PVC blends (mean ± σ, n = 5).

A profile of plasticizer migration tests from PVC blends is shown in Figure 10. The PVC blend disks tested were alternately sandwiched with LDPE disks designed to absorb the leached plasticizer. Each stack was stressed with a 5 kg ballast, and migration was tested at 70 °C in a circulating air dryer. It was observed that the LDPE disks did not absorb the leached DFA-based plasticizer, whereas they showed a greasy surface for the PVC blend. Thus, after a defined time, this sample was wiped, removing the greasy film before weighing.

The migration value of all DFA-based plasticizers is relatively small compared to monomeric plasticizers, such as DEHT in blend that has ~20% loss (Figure 10). It can be observed that the major influence on the migration rate for 28 days is determined by the ratio of DFA to ADA in the plasticizer—see PD 30 in blend versus PD 31 in blend or PD 43 in blend. Initially, at this temperature, for up to two days, all samples showed the same plasticizer loss from the PVC blends. Larger differences between the sample masses were seen after 7 days and after 28 days. PD_31 in blend and PD_43 in blend behaved similarly, while PD_30 in blend showed almost twice as large a plasticizer loss of about 8%. Based on these migration results and previous observations, the apparent phenomenon of PD_30 in blend displaying a greasy surface after 48 h from pressing the pellets was sought to be explained. The problem of migration of the plasticizer to the surface can be explained in this case by the mechanisms of plasticization. Polymer–plasticizer interactions dominate below the critical plasticizer content, and plasticizer–plasticizer interactions dominate at higher plasticizer concentrations, increasing the heterogeneity of the blends [43]. It is generally considered that the compatibility of a particular polymer–plasticizer system is the amount of plasticizer that can be added to the polymer before phase separation occurs. The plasticizer behaves as a solvent for the polymer, and the solubility parameter concept is applied to this “solution“ [43].

The solubility parameter (*δ*) is defined as outlined below:

(4) $$\delta^{2}=\delta_{d}^{2}+\delta_{p}^{2}+\delta_{h}^{2}$$

where

*δ_d_*—the dispersive term;

*δ_p_*—the polar term;

*δ_h_*—the hydrogen-bonding term.

The solubility parameter components could be estimated from group contributions according to the method of Hoftyzer and Van Krevelen using the following equations:

(5) $$\delta_{d}=\frac{\sum F_{di}}{V};\;\delta_{p}=\frac{\sqrt{\sum F_{pi}^{2}}}{V};\;\delta_{h}=\sqrt{\frac{\sum E_{hi}}{V}}$$

where

*F_d_*—dispersion component of the molar attraction function, (MJ/m^3^)^1/2^·mol^−1^;

*F_p_*—polar component of the molar attraction function, (MJ/m^3^)^1/2^·mol^−1^;

*E_h_*—the hydrogen bonding energy per structural group, J·mol^−1^;

*V*—the molar volume of the solvent molecule or the structural unit of the polymer, cm^3^·mol^−1^.

The calculated value of the solubility parameter for PVC according to the method of Hoftyzer and Van Krevelen is 19.7 (MJ/m^3^)^1/2^ [44]. We calculated this value using data tables [44] for DEHT and some DFA-based plasticizer structures based on the obtained average M_w_ (Table 5).

The difference in plasticizer and polymer solubility parameters must be small for good miscibility. A general rule is that the solubility parameter of a good solvent should depart by no more than ±2 (MJ/m^3^)^1/2^ (2 MPa^1/2^) from that of the polymer [44]. Taking into account the calculations presented in Table 5, only one plasticizer molecule with DFA units had a value slightly above 2. Thus, it can be concluded that plasticizer compatibility is minimal if there are such compounds in the blend where the ratio of ADA:DFA units is less than 4:1. Moreover, even in the case of DFA-based plasticizers—for which this value is close enough to the difference in PVC-DEHT solubility parameters—the critical plasticizer content in the blend is another important parameter. Examining the moldings of the blend that were stored at room temperature after a year, it was observed that the T_g_ values have changed by about 1 °C. Such an observation would confirm that the value of 50 phr of plasticizer is too high. By plasticizer–plasticizer interactions, part of the compound is removed from the PVC blend until a stable system is reached, where polymer–plasticizer interactions dominate.

**Figure 11:**
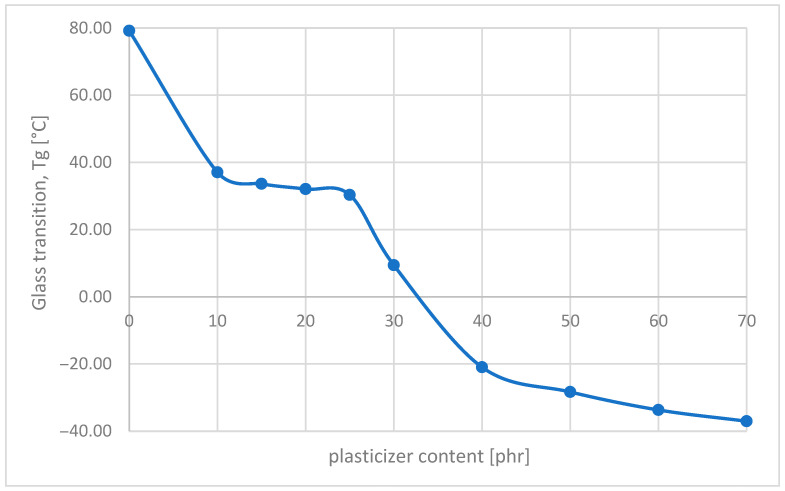
Changes in the T_g_ of PVC plasticized with PD_30.

Based on the literature review, the critical plasticizer content is determined, for instance, from T_g_ values determined with DSC as a cusp in the figure of T_g_ vs. the composition of the PVC–plasticizer. In our study, we determined the T_g_ values of the obtained films by casting from a THF solution with different PD_30 contents: 10, 15, 20, 25, 30, 40, 50, 60, and 70 phr. As mentioned earlier, the plasticization of the polymer and plasticizer, representing compatibility, is typically observed by a shift in the T_g_ of the polymer. In this case, the addition of 10 phr (9 wt%) of oligoester caused a decrease in the T_g_ of 42 °C, confirming the plasticizing ability of PVC by this compound. The content of 15 (13 wt%) to 25 phr (20 wt%) of oligoester already slightly changes T_g_ by the next 6 °C (Figure 11).

In the range from 25 phr (20 wt%) to 40 phr (28 wt%) of plasticizer, there is the greatest change in T_g_. For the critical plasticizer value, the cusp point is about 30 phr (23 wt%). Polymer–plasticizer interactions dominate below the critical plasticizer content [40]. In the case of PVC plasticized with DEHP, the critical plasticizer content is ~30 wt% of DEHP. Thus, in the range above 25 phr and below 30 phr, PVC blends with this plasticizer represent the most homogeneous structure.

## 4. Conclusions

This paper describes novel oligoesters based on saturated dimerized fatty acids (DFA) obtained as potential biobased PVC plasticizers in a two-step, one-pot process. They were synthesized by polyesterification of DFA and adipic acid (ADA) with triethylene glycol (TEG) using different DFA to ADA molar ratios of 1:4 and 1:9. The ends of the oligoester chain formed were capped with 2-ethylhexanol, added after the required parameters were reached. The introduction of the catalyst (0.2 wt% Fascat 4100) in two portions accelerated the oligoester chain formation throughout the entire process. Furthermore, it was observed that the DFA must be introduced into the reaction system from the beginning of the reaction, otherwise, it precipitates out of the product over time.

Based on the preliminary testing method (PVC film casting), it was possible to eliminate incompatible compounds and focus on those with properties closest resembling commercial samples, thus showing the best promise of being a viable alternative. Oligoesters, whose PVC films showed similar properties to those obtained with DEHT, were selected for the preparation of the PVC dry blends. Dry blends of S-PVC were made with both the selected oligomers PD_30 (DFA:ADA = 1:4, 1.10 [-OH]), PD 31 (DFA:ADA = 1:9, 1.10 [-OH]), PD 43 (DFA:ADA = 1:9, 1.35 [-OH]) and the commercial plasticizers (DEHT and DEHA) in the amount of 50 phr. Additionally, calcium carbonate—10 phr and thermal stabilizer Ca/Zn—4.5 phr were used.

The best physicochemical and mechanical properties were achieved for oligoester PD 43. The use of the 1:9 molar ratio of DFA:ADA, and an excess of 2-EH in the synthesis, made it possible to obtain a plasticizer with an average molecular weight of 2710 Da, a good viscosity of 420 mPa·s, and the best compatibility with PVC among those synthesized. Plasticizer PD_43 also featured the lowest migration from the PVC blends. The PVC blend obtained with this plasticizer had the lowest hardness of 88 ShA among the new plasticizers tested. It also had the highest tensile strength of 18.3 MPa, better than PVC blends obtained with the commonly used monomeric plasticizers DEHT and DEHA, and a comparable elongation at break (317%) and Young’s modulus (17 MPa).

The amount of 50 phr proved to be too high for the PD 30 plasticizer, resulting in an oily layer on the molding of the blend. A solubility parameter calculated for this composition explains the slightly lower compatibility of this oligoester with S-PVC. In addition, the critical plasticizer content (determined based on T_g_, below which polymer–plasticizer interactions dominate) and the range in plasticizer content that most effectively plasticize S-PVC were lower than 50 phr.

Based on these results, it can be concluded that PD 43 could be used as a primary plasticizer (in the amount of 50 phr) for obtaining blends from S-PVC. Other oligoesters obtained (e.g., PD 30) can also be used as plasticizers but in quantities of less than 50 phr or in combination with another plasticizer. Furthermore, mixing the proposed plasticizers with others, e.g., monomeric ones, can result in more flexible materials and is an interesting avenue for further research. Overall, with further testing, PD 43 may present an environmentally friendly, sustainable, and safe alternative to the commercial PVC plasticizers used in the industry today.

## Figures and Tables

**Table 1:** Components used for the synthesis of plasticizers and a measure of the progress of a reaction.

Sample Code	Molar Ratios of Acids DFA:ADA	Summary nOHnCOOH Based on Equation (2)	Reagents [mol]	Cat. Fascat 4100 [wt%]	AV on the End of Step [mg KOH/g]	Time of Step [h]	Other Information
**PD_03**	100% DFA	1.12	DFA 0.25 TEG 0.24 2-EH 0.09	0.20	0.8	9	synthesis in 500 mL flask; clear product
**PD_13**	100% ADA	1.15	I ADA 0.44 TEG 0.42	I 0.20	I 39.0 II 1.6	I 3 II 6	precipitate after 2 years
			II 2EH 0.17				
**PD_14**	1:9	1.15	I ADA 0.42 TEG 0.44	I 0.15 II 0.05	I 62.6 II 30.9 III 1.8	I 1 II 7 III 7	slightly cloudy product; precipitate after 2–3 months
			II DFA 0.05				
			III 2-EH 0.19				
**PD_17**	1:9	1.35	I ADA 0.42 TEG 0.44 DFA 0.05	I 0.20	I 30.6 II 1.1	I 5 II 5	excess 2EH was distilled
			II 2EH 0.37				
**PD_30**	1:4	1.10	I ADA 1.25 TEG 1.47 DFA 0.31	I 0.15 II 0.05	I 31.6 II 0.9	I 7 II 10	synth. in 1000 mL flask; larger scale = longer synth. time
			II 2EH 0.49				
**PD_31**	1:9	1.10	I ADA 2.50 TEG 2.62 DFA 0.28	I 0.15 II 0.05	I 42.0 II 1.0	I 9 II 17	synth. in 2000 mL flask; larger scale required for dry-blend production
			II 2-EH 0.87				
**PD_43**	1:9	1.35	I ADA 2.08 TEG 2.18 DFA 0.23	I 0.10 II 0.10	I 41.4 II 0.7	I 7.5 II 4.5	the same as PD_17 but on a larger scale—2000 mL; excess 2EH was distilled
			II 2EH 1.88				

**Table 2:** Properties of the obtained plasticizers.

Sample Code	Molar Ratios of Acids DFA:ADA	Product Properties						
		Molecular Weight (SEC)			Viscosity 25 °C [mPa·s]	Density 25 °C [g/cm^3^]	Color Scales	
		${\bar{M}}_{n}$ [g/mol]	${\bar{M}}_{w}$ [g/mol]	Ð			Hazen	Iodine
**PD_03**	100% DFA	2780	4720	1.7	2860	0.96	448	2.9
**PD_13**	100% ADA	1460	2490	1.7	380	1.10	146	0.7
**PD_14**	1:9	1430	2580	1.8	570	1.06	795 (precipitate)	5.3
**PD_17**	1:9	1020	1840	1.8	260	1.04	401	2.4
**PD_30**	1:4	2640	4760	1.8	2090	1.05	340	3.0
**PD_31**	1:9	2130	3840	1.8	1100	1.08	742	5.4
**PD_43**	1:9	1590	2710	1.7	420	1.06	232	1.8

**Table 3:** Properties of the PVC films—the preliminary test of compatibility.

PVC Film Code	Transparency	Flexibility (Can the PVC Film Be Easily Bent?)	Is the Surface of the Film Dry?	T_g_ [°C]	Qualificate to PVC Blends?
**PVC_film** **without plasticizer**	− white color	− rigid	+	80	comparison sample
**DEHT_film**	+	+	+	−14	comparison sample
**PD_03_film**	−	− rigid	− greasy surface	79	− opaque film with white discoloration
**PD_13_film**	+/− partly opaque	+/− partly rigid	− greasy after a few days	−25	− loses its positive properties after time
**PD_14_film**	+	+	+	−30	− precipitate in plasticizers
**PD_17_film**	+	+	+	−29	+ but there is too much 2EH in plasticizer = low Mw
**PD_30_film**	+	+	+	−28	+ slight shrinkage of the film after drying
**PD_31_film**	+	+	+	−27	+ slight shrinkage of the film after drying
**PD_43_film**	+	+	+	−28	+ the same as PD_17_film

**Table 4:** Comparison of the thermal properties of the obtained PVC blends.

	T_g_ [°C]	Temperature [°C]		
		Mass Loss of 5%	1st Stage Decomposition	2nd Stage Decomposition
**PD_30 in blend**	nd *	281	300	466
**PD_31 in blend**	−25	270	300	463
**PD_43 in blend**	−27	261	300	460
**DEHT in blend**	−16	265	286	475
**DEHA in blend**	−35	238	283	469

nd *—not designated.

**Table 5:** The calculated solubility parameter values based on hypothetical assumed molar ratios of DFA:ADA, general formula of oligoester, and Formulas (4) and (5).

Plasticizer	${\bar{M}}_{w},$		The General Formula of Oligoester	Molar Ratio of DFA:ADA	δ, (MJ/m^3^)^1/2^	δpolymer–δplasticizer
	SEC	cal.				
**DEHT**	390		-	-	17.93	1.77
**PD_30**	4760	4850	2-EH-(ADA-TEG)_13_-DFA-TEG-DFA-2-EH	1:6.5	18.02	1.68
		4750	2-EH-(ADA-TEG)_10_-(DFA-TEG)_2_-DFA-2-EH	1:3.3	17.78	2.02
**PD_31**	3840	3810	2-EH-(ADA-TEG)_9_-DFA-TEG-DFA-2-EH	1:4.5	17.87	1.83
		3910	2-EH-(ADA-TEG)_12_-DFA-2-EH	1:12	18.13	1.57
**PD_43**	2700	2610	2-EH-(ADA-TEG)_7_-DFA-2-EH	1:7	17.97	1.73
		2870	2-EH-(ADA-TEG)_8_-DFA-2-EH	1:8	18.02	1.68
		3130	2-EH-(ADA-TEG)_9_-DFA-2-EH	1:9	18.07	1.63

## Data Availability

The original contributions presented in this study are included in the article. Further inquiries can be directed to the corresponding author.
